# Disparity of Hepatocellular Carcinoma in Tumor Microenvironment-Related Genes and Infiltrating Immune Cells between Asian and Non-Asian Populations

**DOI:** 10.3390/genes12081274

**Published:** 2021-08-20

**Authors:** Lien-Hung Huang, Ting-Min Hsieh, Chun-Ying Huang, Yueh-Wei Liu, Shao-Chun Wu, Peng-Chen Chien, Ching-Hua Hsieh

**Affiliations:** 1 Department of Neurosurgery, Kaohsiung Chang Gung Memorial Hospital and Chang Gung University College of Medicine, Kaohsiung 88301, Taiwan; ahonbob@gmail.com; 2 Department of Trauma Surgery, Kaohsiung Chang Gung Memorial Hospital and Chang Gung University College of Medicine, Kaohsiung 88301, Taiwan; hs168hs168@gmail.com (T.-M.H.); junyinhaung@yahoo.com.tw (C.-Y.H.); VENU_CHIEN@hotmail.com (P.-C.C.); 3 Department of General Surgery, Kaohsiung Chang Gung Memorial Hospital and Chang Gung University College of Medicine, Kaohsiung 88301, Taiwan; anthony0612@adm.cgmh.org.tw; 4 Department of Anesthesiology, Kaohsiung Chang Gung Memorial Hospital and Chang Gung University College of Medicine, Kaohsiung 88301, Taiwan; shaochunwu@gmail.com

**Keywords:** hepatocellular carcinoma, tumor microenvironment, Asian, non-Asian, ESTIMATE, CIBERSORT

## Abstract

Hepatocellular carcinoma (HCC) is the most common cause of primary liver cancer deaths worldwide. The major risk factors for liver cancer development are cirrhosis, hepatitis B virus (HBV), hepatitis C virus (HCV) infection, and chronic alcohol abuse. HCC displays heterogeneity in terms of biology, etiology, and epidemiology. In Southeast Asia and Africa, chronic HBV infection is a major risk factor for HCC, whereas chronic HCV infection is a risk factor for HCC in western countries and Japan. Environmental and genetic conditions also play a role in the regional and temporal variations in the incidence of HCC. In this study, we used the ESTIMATE (ESTIMATE, Estimation of stromal and immune cells in malignant tumor tissues using expression data) algorithm and the CIBERSOFT tool to analyze gene expression profiles and infiltrating immune cells in HCC between Asian and non-Asian patients. The results showed that stromal and immune scores were dependent on overall survival (OS) in non-Asian patients but not in Asian patients. Kaplan–Meier survival analysis revealed four differentially expressed genes (DEGs) that were significantly associated with OS in non-Asian patients only. CIBERSORT (CIBERSORT, Cell type identification by estimating relative subsets of known RNA transcripts) analysis indicated that the composition of infiltrating immune cells was significantly different between Asian and non-Asian patients. By parsing the subclasses of HCC, the ability to predict prognosis and guide therapeutic targets for potentially actionable HCC may be improved.

## 1. Introduction

HCC accounts for the majority of primary liver cancers, representing approximately 90% of all cases of primary liver cancer [1,2]. The major risk factors for liver cancer development are cirrhosis, hepatitis B virus (HBV), hepatitis C virus (HCV) infection, and chronic alcohol abuse [3]. Other risk factors, such as aflatoxin B1 intake [4], metabolic syndrome [5], and genetic conditions [6], are well-characterized contributors to liver carcinogenesis.

HCC is an inflammation-related cancer characterized by persistent inflammation in the absence of infectious agents or exposure to toxic compounds. An aberrant microenvironment plays an important role in the development of HCC. The microenvironment of HCC consists of extracellular matrix components, growth factors, cytokines, and stromal cells, such as inflammatory cells, carcinoma-associated fibroblasts (CAFs), hepatic stellate cells (HSCs), tumor-associated macrophages (TAM), endothelial cells, pericytes, dendritic cells, and stem/progenitor cells [3,7,8].

CAFs are one of the most crucial cell types in the stroma and play a role in HCC growth, invasion, angiogenesis, and metastasis [9]. CAFs secrete VEGF, PDGF, FGF, and SDF-1 to recruit endothelial progenitor cells and promote angiogenesis [10]. Through matrix metalloproteinase (MMP) secretion, CAFs facilitate extracellular matrix remodeling and tumor invasion [11]. CAFs stimulate monocytes and macrophage infiltration through cytokine and chemokine secretion [12]. HSCs act as effectors during liver injury. In viral infections, hepatotoxicity, or HCC, HSCs trans-differentiate into activated myofibroblast-like cells and infiltrate the site of the injury [13]. Activated HSCs form pro-angiogenic and pro-inflammatory microenvironments by secreting diverse cytokines, growth factors, and extracellular matrix component proteins. Infiltration of activated HSCs into the stroma modulates endothelial cell and hepatocyte proliferation and leads to stromal remodeling [14]. HSCs also provide an immunosuppressive niche through the induction of regulatory T cells and myeloid-derived suppressor cells (MDSCs) in HCC [15,16].

In addition to stromal cells, various immune cells are present within the tumor microenvironment in HCC. These diverse cells communicate with each other through cytokine and chemokine production [17]. TAMs are the main type of inflammatory cells and resemble M2-polarized macrophages in the tumor microenvironment [18]. TAMs release a number of factors that regulate cancer cell proliferation, migration, invasion, angiogenesis, metastasis, and survival [19]. The level of circulating regulatory T cells (Tregs) increases and is correlated with HCC progression. Tregs suppress CD8^+^ T-cell cytolytic molecule release and production. The proliferation, cytokine production, and activation of CD8^+^ T cells are repressed by Tregs [20].

Crosstalk between stromal and immune cells in the tumor microenvironment is involved in cancer development. On the basis of the gene expression of stromal and immune cells in the Cancer Genome Atlas (TCGA) database, estimation of stromal and immune cells in malignant tumor tissues using expression data (ESTIMATE) can be used to score stromal and immune components in the tumor tissue [21]. The stromal and immune scores correlate with tumor progression and patient survival in several tumor types, including breast cancer [22], renal cell carcinoma [23], gastric cancer [24], lung cancer [25], and liver cancer [26].

The incidence rates of HCC are highest in Asia Pacific, East and Southeastern Asia, and central sub-Saharan Africa and lowest in southern Latin America and tropical Latin America [27,28]. The high prevalence of HBV infection is the leading cause of HCC in Asia and sub-Saharan Africa. In North America, Europe, and Japan, HCV infection is one of the main causes of HCC [2,29,30]. Excessive alcohol intake is a leading risk factor for HCC in the United States and Europe [31]. In the United States, 50–60% of HCC patients are infected with HCV, 36.6% have some features of metabolic syndrome, 20–25% have alcoholic liver disease, and 10–15% are infected with HBV [32,33]. In China, HBV infection contributes to 63.9% of HCC cases, 27.7% of HCC patients are infected with HCV, 25% are exposed to aflatoxin, and 15.7% suffer from alcoholism [34]. In India, HBV infection contributes to 50–70% of HCC cases, 10–15% of HCC patients are infected with HCV, and 6–16% have heavy alcohol intake [35,36].

The environment, genetic conditions, and etiology could play a role in the regional and temporal variations in the incidence of HCC. In this study, we used ESTIMATE and CIBERSOFT to analyze and compare the gene expression profiles of tumor microenvironment-related genes and infiltrating immune cells in HCC between Asian and non-Asian patients.

## 2. Materials and Methods

### 2.1. Data Acquisition

The clinical profiles and the fragments per kilobase of transcript per million (FPKM) of the RNA sequence data in HCC patients were obtained from TCGA database (Project ID, “TCGA-LIHC” (https://gdc.nci.nih.gov/, accessed on 26 June 2020). The cases with missing clinical data were excluded, and the final sample size was 359 (205 Asian patients and 154 non-Asian patients). The immune scores, stromal scores, and estimate scores were calculated by applying the ESTIMATE algorithm, in which R package = estimate (version 1.0.13) (https://bioinformatics.mdanderson.org/estimate/rpackage.html, accessed on 1 August 2020).

### 2.2. Definition of Differentially Expressed Genes (DEGs)

On the basis of the median values of the immune and stromal scores, patients with HCC were categorized into high and low-score groups. Data analysis was conducted using the limma R package (version 3.46.0). Genes with a *p* value of <0.05 and |fold change| > 1.5 were defined as differentially expressed genes (DEGs).

### 2.3. Protein–Protein Interaction (PPI) Network

The PPI network of DEGs was acquired using the Search Tool for Retrieval of Interacting Genes (STRING, https://string-db.org/, version11.0b, accessed on 26 April 2021) online database [37].

### 2.4. Immune Cell Profiling

The CIBERSOFT method (https://cibersort.stanford.edu/, accessed on 23 April 2021) was used to evaluate the relative proportions of 22 tumor-infiltrating immune cell profiles on the basis of expression files, including B cells, T cells, natural killer cells, macrophages, dendritic cells, and myeloid subsets. The abundance of member cell types in a mixed cell population was estimated using gene expression data.

### 2.5. Survival Analysis

The relationship between overall survival and gene expression levels of DEGs was analyzed using the Kaplan–Meier method. In the univariate analysis, the effects of each DEG on OS were examined using the log-rank test. Hazard ratios were used to analyze the survival effect of immune cell profiling using CIBERSOFT. A survival analysis was performed using the R package survival (version 3.2.7) and survminer (version 0.4.8).

### 2.6. Gene Ontology and Kyoto Encyclopedia of Genes and Genomes (KEGG) Enrichment Analysis

The R package clusterProfiler (version 3.18.1) was used for the gene ontology and KEGG enrichment analysis of DEGs.

## 3. Results

### 3.1. Study Patients’ Demographics and Kaplan–Meier Survival Analysis

**Figure 1:**
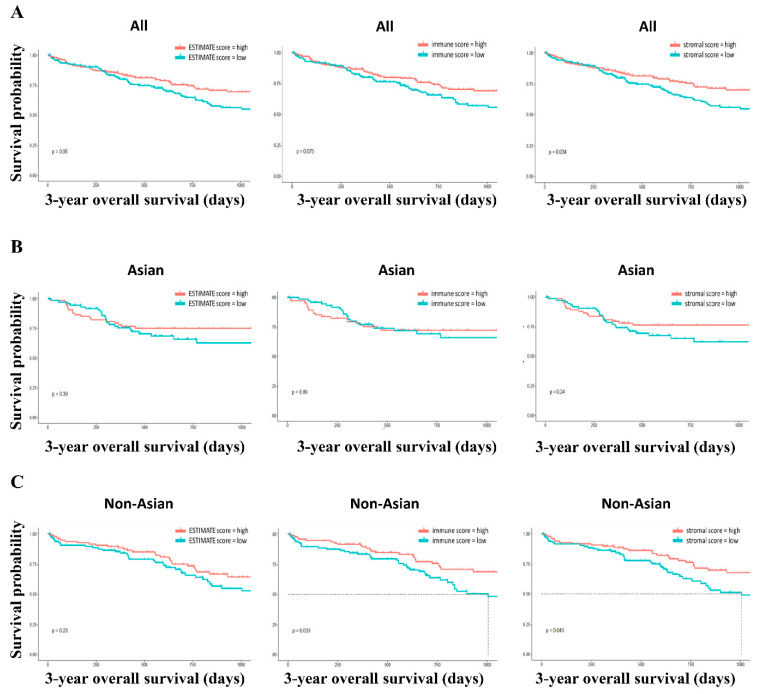
Kaplan–Meier survival curves of high- and low-score groups for 3-year OS in all (**A**), Asian (**B**), and non-Asian (**C**) patients.

The data of a total of 359 HCC patients, including 205 Asian and 154 non-Asian patients, were obtained from TCGA database. The clinical information of these patients is presented in Table 1. Compared with non-Asian patients, Asian patients were younger, thinner, more frequently male, and had a lower survival rate. Using the ESTIMATE algorithm, stromal and immune scores were calculated. On the basis of the median value of these scores, patients with HCC were divided into high- and low-score groups. Kaplan–Meier survival curves were used to assess the potential connection between 3-year overall survival (OS) and the ESTIMATE/stromal/immune scores for HCC (Figure 1). HCC patients with high stromal scores had better OS than those with low stromal scores (*p* = 0.034). In terms of ESTIMATE and immune scores, although patients with high scores showed longer OS than those with low scores, the difference was not statistically significant (*p* = 0.06 in ESTIMATE scores and *p* = 0.075 in immune scores) (Figure 1A). In Asian patients, the Kaplan–Meier survival curves showed no statistically significant difference between the high- and low-score groups of the ESTIMATE/stromal/immune scores (Figure 1B). In non-Asian patients, the high immune score group and the high stromal score group had better OS than the low immune score group (*p* = 0.033 for the immune score and *p* = 0.043 for the stromal score, Figure 1C).

### 3.2. Screening of DEGs

**Figure 2:**
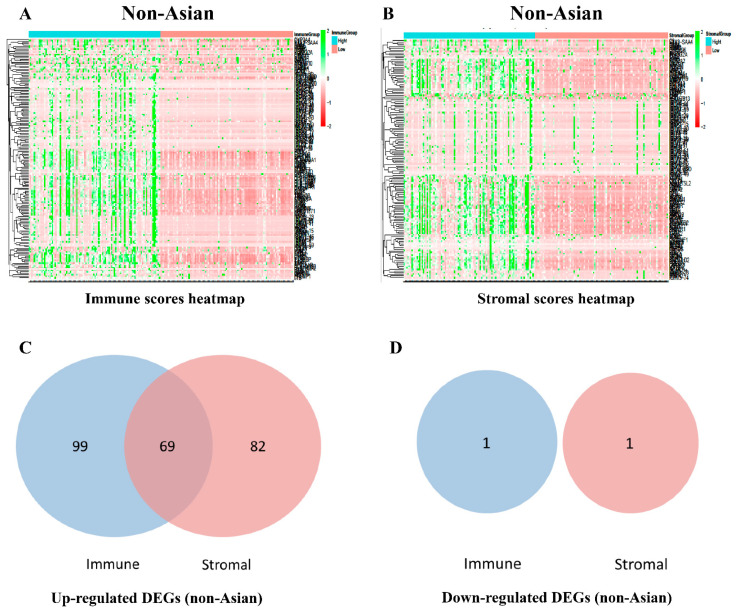
Comparison of gene expression profiles in non-Asian patients with HCC. Heatmaps of the unique gene expression profiles based on immune scores (**A**) and stromal scores (**B**). Highly expressed genes are shown in green, and genes with low expression levels are shown in red. Venn diagrams of the numbers of up-regulated (**C**) or down-regulated (**D**) common DEGs in the stromal and immune score groups.

**Figure 3:**
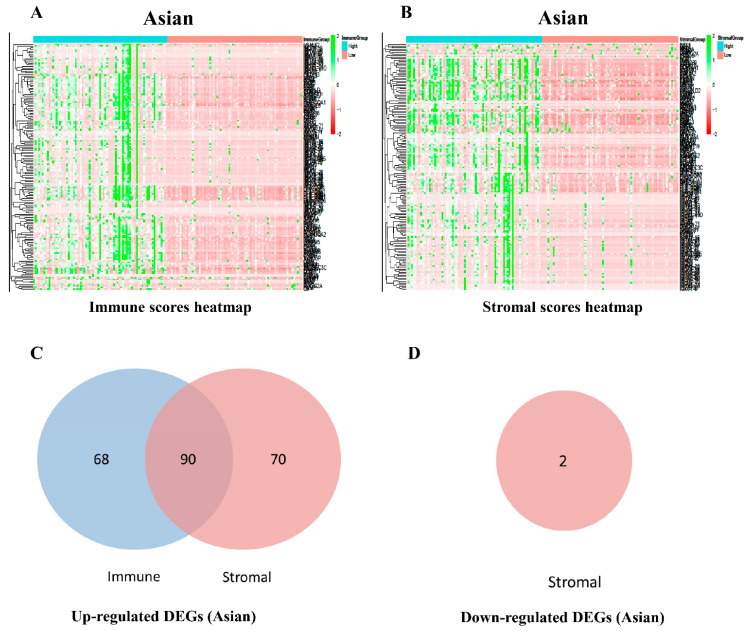
Comparison of gene expression profiles in Asian patients with HCC. Heatmaps of the unique gene expression profiles based on immune scores (**A**) and stromal scores (**B**). Highly expressed genes are shown in green, and genes with low expression levels are shown in red. Venn diagrams of the numbers of up-regulated (**C**) or down-regulated (**D**) common DEGs in the stromal and immune score groups.

According to the criteria of |fold change| > 1.5 and adjusted *p* value < 0.05, we compared the high and low immune score groups in non-Asian HCC patients. A total of 169 genes were significantly differentially expressed, including 168 upregulated genes and 1 downregulated gene (Supplementary Table S1). In the stromal score groups, 151 upregulated genes and 1 downregulated gene were identified (Supplementary Table S2). The unique gene expression profiles are displayed in a heatmap (Figure 2A,B). Venn diagrams showed 69 intersecting DEGs (Figure 2C,D). In Asian patients with HCC, we identified 158 upregulated genes in the immune score group. In the stromal score groups, 160 upregulated genes and 2 downregulated genes were identified (Figure 3 and Supplementary Tables S3 and S4). A total of 90 DEGs are displayed in Figure 3, although the differences between Asian patients in the high- and low-scoring groups of ESTIMATE, stromal, and immune scores were not statistically significant.

### 3.3. Gene Ontology and KEGG Enrichment Analysis of DEGs

**Figure 4:**
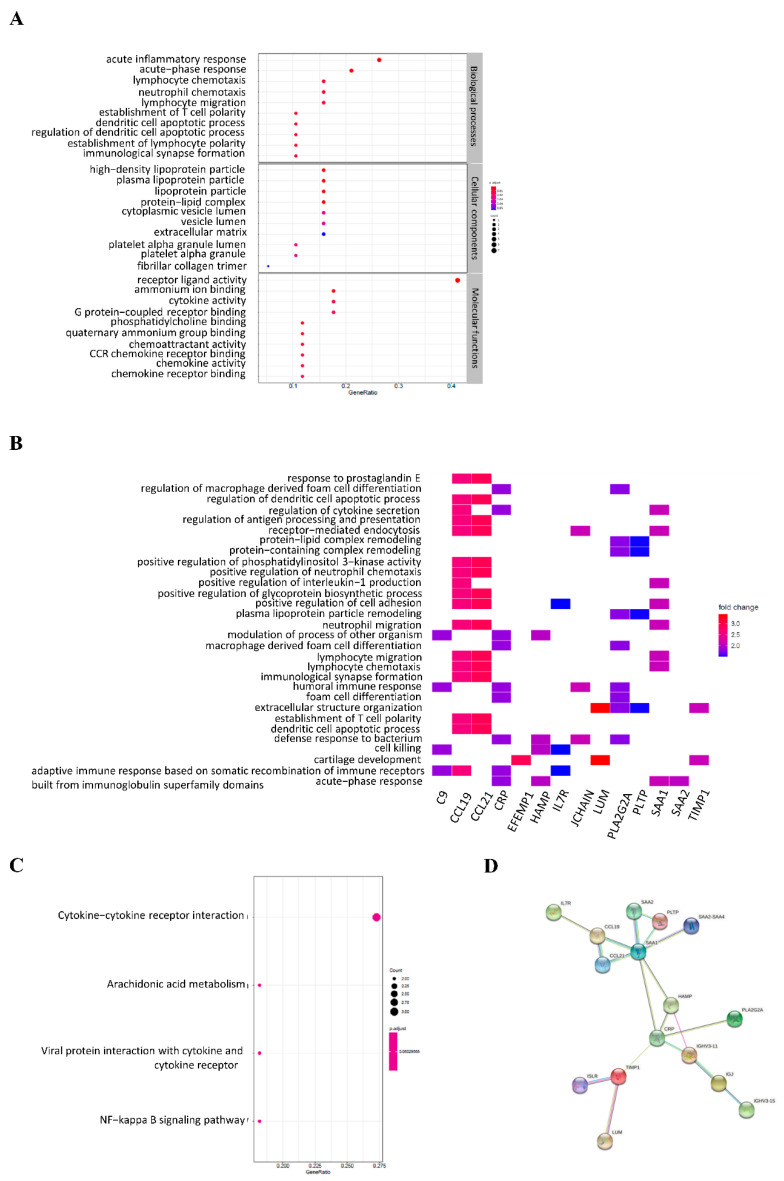
Functional enrichment analysis of differentially expressed genes in non-Asian patients with HCC. GO terms (**A**), GO heat-map (**B**), KEGG pathways (**C**), and PPI network (**D**) for the DEGs.

**Figure 5:**
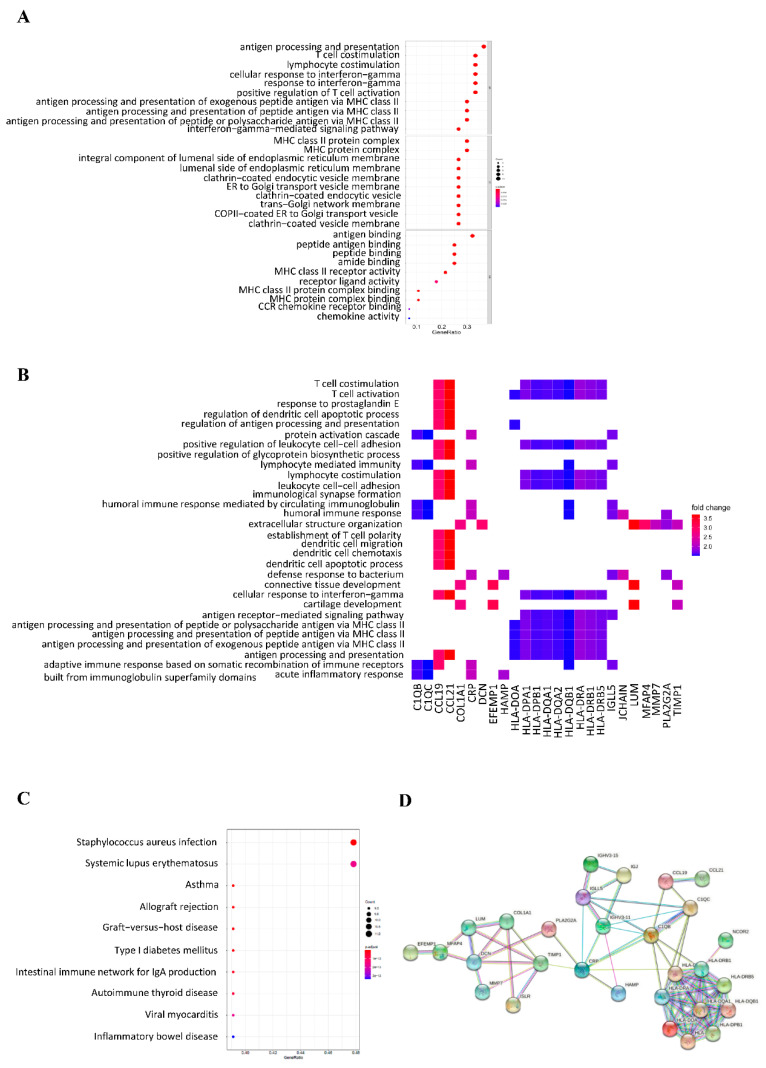
Functional enrichment analysis of differentially expressed genes in Asian HCC patients. GO terms (**A**), GO heat-map (**B**), KEGG pathways (**C**), and PPI network (**D**) for the DEGs.

The gene ontology analysis showed that the DEGs were mainly involved in the immune response in HCC. In the non-Asian group, DEGs were significantly enriched in the acute inflammatory response, acute phase response, lymphocyte chemotaxis, neutrophil chemotaxis, and lymphocyte migration in biological processes (Figure 4A). The GO heat plot shows the relationship between the genes and terms of biological processes (Figure 4B). The genes involved in the KEGG pathway enrichment results for DEGs were mainly involved in cytokine–cytokine receptor interactions, viral protein interactions with cytokine and cytokine receptors, and the NF-kappa B signaling pathway (Figure 4C). In the Asian group, DEGs were significantly enriched in antigen processing and presentation, T cell co-stimulation, lymphocyte co-stimulation, cellular response to interferon-γ, and positive regulation of T cell activation (Figure 5A). The GO heat plot shows the relationship between the genes and the terms of biological processes (Figure 5B). The KEGG pathway enrichment results for DEGs were mainly associated with Staphylococcus aureus infection and systemic lupus erythematosus (Figure 5C).

### 3.4. PPI Network of DEGs

The network interaction analysis of DEGs was performed using the online STRING tool. In the non-Asian group, a total of 19 edges and 16 nodes were formed in the network, including IL7R, CCL19, CCL21, SAA1, SAA2, SAA2-SAA4, PLTP, CRP, HAMP, PLA2G2A, IGHV3-11, IGJ, IGHV3-15, TIMP1, ISLR, and LUM (Figure 4D). In the Asian group, a total of 78 edges and 29 nodes were formed in the network, including EFEMP1, MFAP4, LUM, DCN, MMP7, COL1A1, TIMP1, ISLR, PLA2G2A, CRP, HAMP, IGHV3-11, IGLL5, IGJ, IGHV3-15, C1QB, C1QC, CCL19, CCL21, HLA-DOA, HLA-DPA1, HLA-DPB1, HLA-DQA1, HLA-DQA2, HLA-DQB1, HLA-DRA, HLA-DRB1, and HLA-DRB5 (Figure 5D).

### 3.5. Hub Genes in HCC

**Figure 6:**
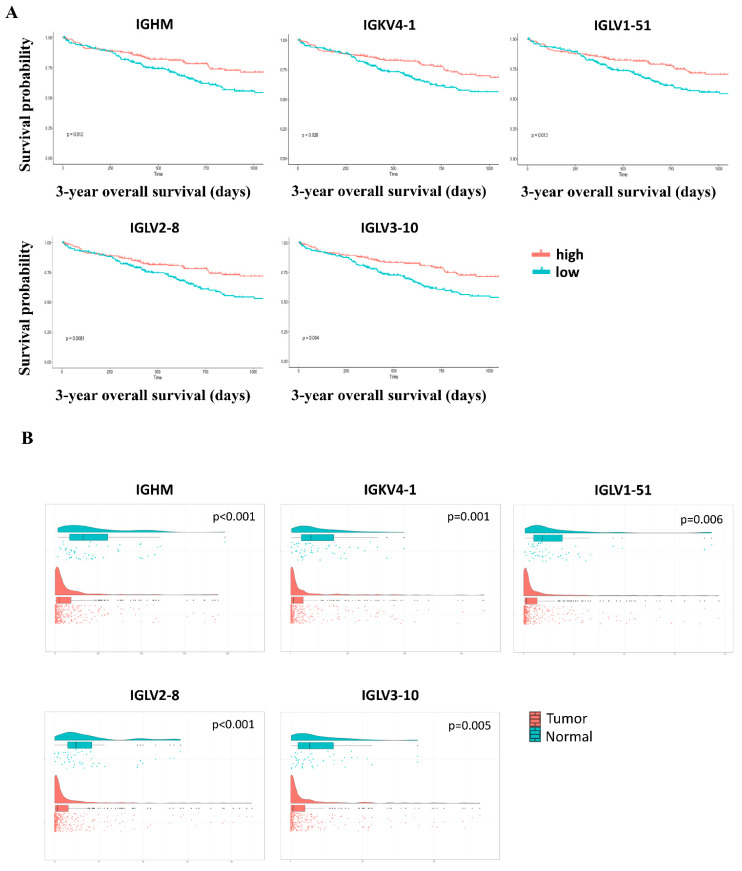
DEGs in HCC. (**A**) Kaplan–Meier survival curves of high and low gene expression groups for 3-year OS. (**B**) The comparison of DEG expression in adjacent normal tissues and tumors.

To identify the hub genes in HCC, 69 DEGs from the non-Asian group were intersected with 90 DEGs from the Asian group, and a total of 61 intersecting genes were analyzed using the Kaplan–Meier survival method. Five genes were significantly associated with OS in patients with HCC: IGHM, IGKV4-1, IGLV1-51, IGLV2-8, and IGLV3-10 (Figure 6A and Table 2). Compared with adjacent normal tissues, these genes showed higher expression in tumors (Figure 6B). We found that these genes were significantly associated with OS in the non-Asian group but not in the Asian group (Table 2).

### 3.6. Immune Cell Profiling and Prognostic Effects

**Figure 7:**
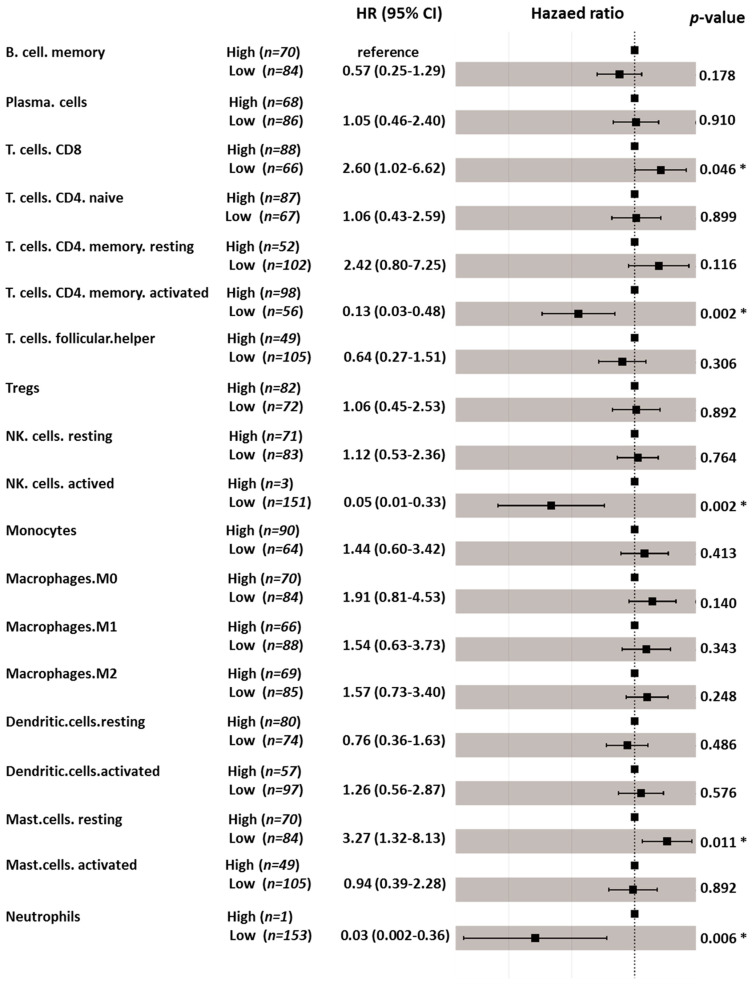
Prognostic effects of individual tumor-infiltrating immune cells subsets on overall survival in Asian patients. * indicates *p* < 0.05.

**Figure 8:**
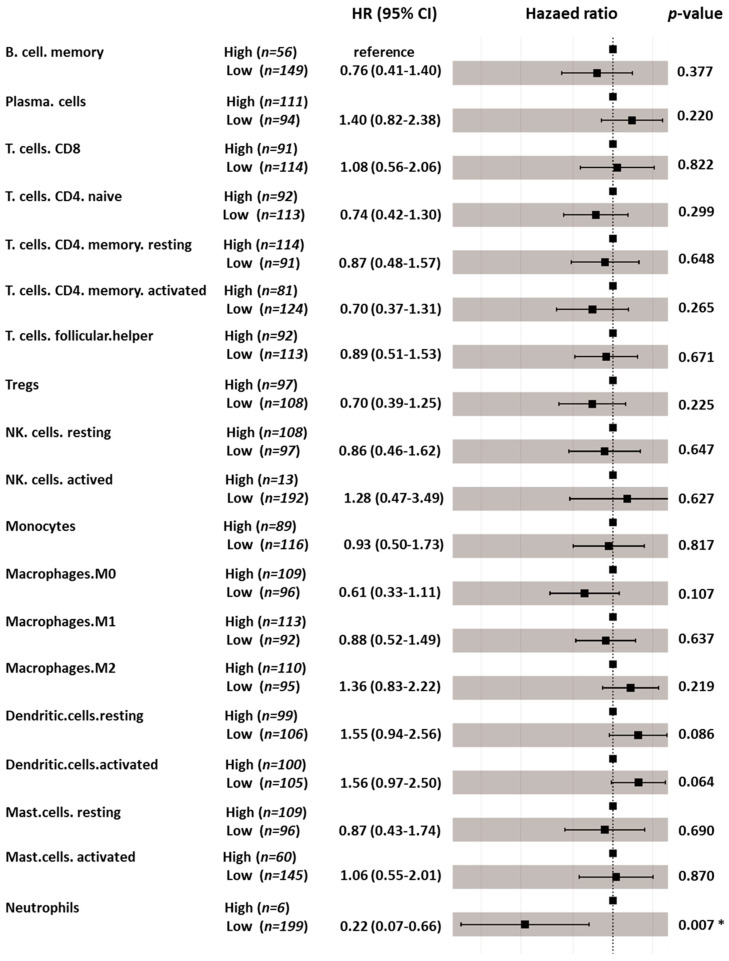
Prognostic effects of individual tumor-infiltrating immune cells subsets on overall survival in non-Asian patients. * indicates *p* < 0.05.

**Figure 9:**
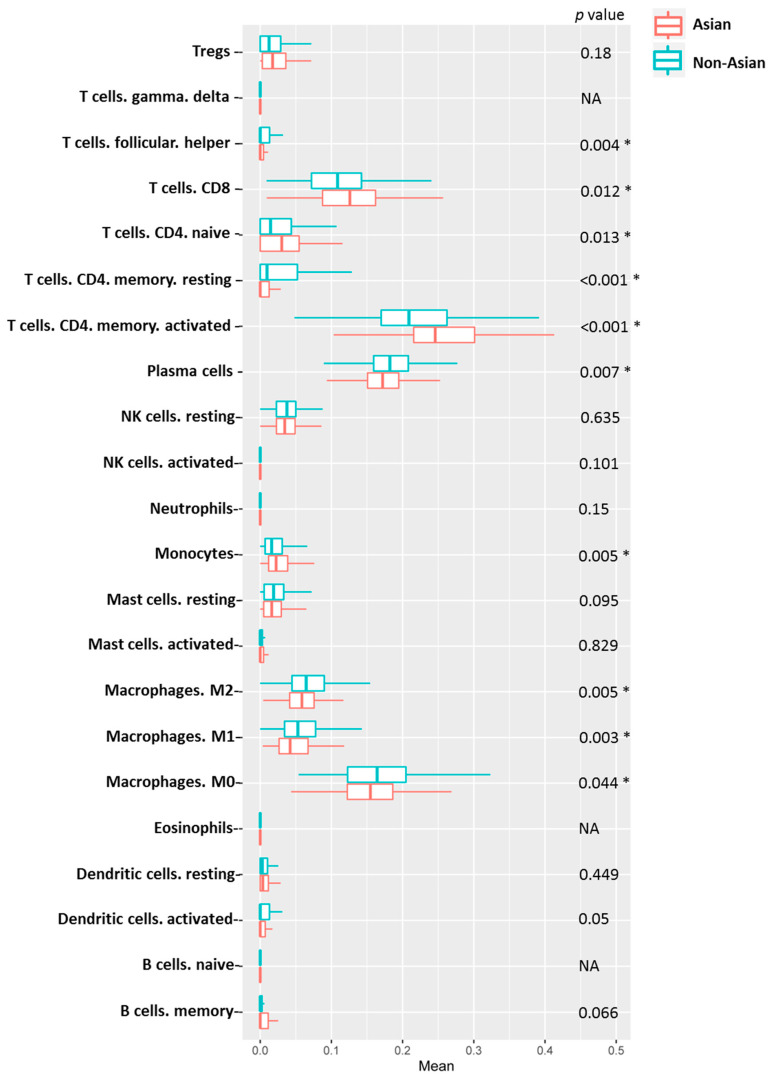
Distribution of tumor-infiltrating immune cell subsets in Asian and non-Asian patients. * indicates *p* < 0.05.

**Figure 10:**
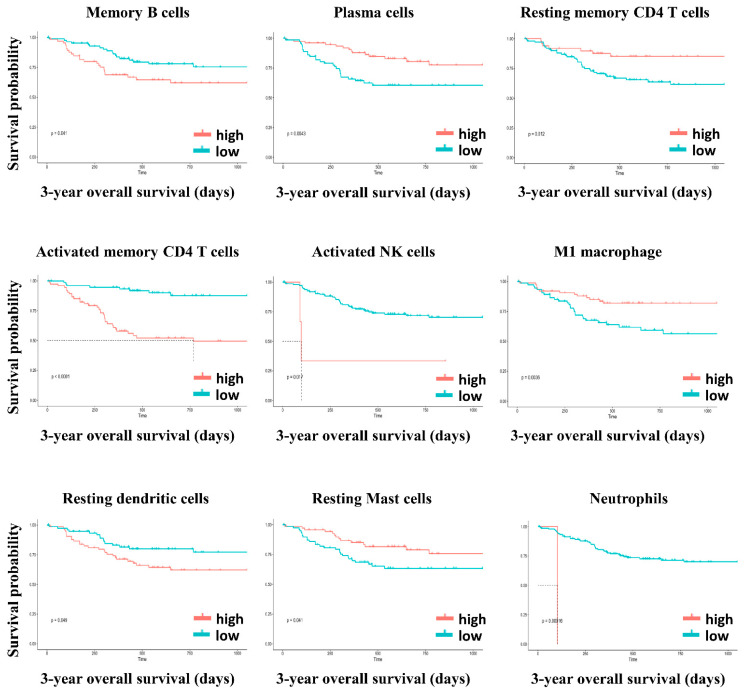
Kaplan–Meier survival curves of high and low levels of tumor-infiltrating immune cells for 3-year OS in Asian patients.

Five immunoglobulin genes showed differential expression in adjacent normal/tumor tissues and were significantly associated with OS. Four of the five genes displayed a significant association with OS in non-Asian patients but not in Asian patients (Table 2). The ESTIMATE algorithm also showed that a higher immune score contributed to a more favorable prognosis in non-Asian patients with HCC. Subsequently, we applied CIBERSORT to estimate the composition of tumor-infiltrating immune cells in Asian and non-Asian patients. The hazard ratios (HRs) and 95% confidence intervals (CIs) of the 19 tumor-infiltrating immune cells are shown in Figure 7 and Figure 8. In Asian patients, CD8 T cells (HR = 2.60, 95% CI = 1.02–6.62; *p* = 0.046), activated memory T cells (HR = 0.13, 95% CI = 0.03–0.48; *p* = 0.002), activated NK cells (HR = 0.05, 95% CI = 0.01–0.33; *p* = 0.002), resting mast cells (HR = 3.27, 95% CI = 1.32–8.13; *p* = 0.011), and neutrophils (HR = 0.03, 95% CI = 0.002–0.36; *p* = 0.006) were significantly associated with OS (Figure 7). In non-Asian patients, only neutrophils (HR = 0.22, 95% CI = 0.07–0.66; *p* = 0.007) were significantly associated with OS (Figure 8). The composition of infiltrating immune cells was significantly different between Asian and non-Asian patients (Figure 9). Compared with non-Asian patients, the compositions of CD8 T cells, naïve CD4 T cells, activated memory CD4 T cells, and monocytes were higher in Asian patients. The compositions of T helper cells; resting memory CD4 T cells; plasma cells; and M0, M1, and M2 macrophages were lower in Asian patients. The Kaplan–Meier survival analysis of the infiltrating immune cell subsets is shown in Figure 10 and Table 3.

**Figure 11:**
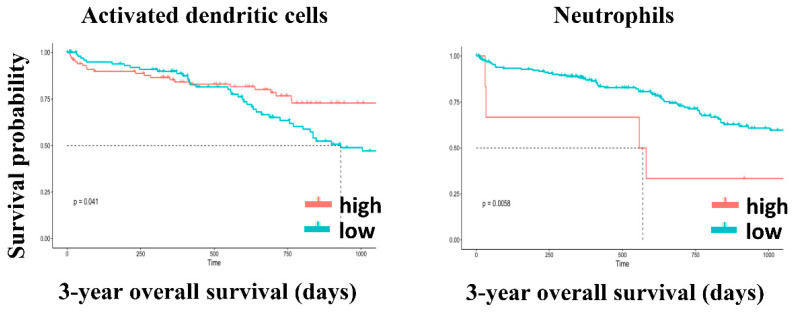
Kaplan–Meier survival curves of high and low levels of tumor-infiltrating immune cells for 3 years OS in non-Asian patients.

In Asian patients, high levels of plasma cells, resting memory CD4 T cells, M1 macrophages, and resting mast cells were significantly associated with longer OS. By contrast, high levels of memory B cells, activated memory CD4 T cells, activated NK cells, resting dendritic cells, and neutrophils were significantly associated with poor OS (Figure 10). In non-Asian patients, high levels of activated dendritic cells were significantly associated with longer OS, and neutrophils were significantly associated with poor OS (Figure 11).

## 4. Discussion

In this study, we used the ESTIMATE algorithm to determine whether stromal and immune scores were associated with OS in patients with HCC. The results showed that only stromal scores were associated with OS. However, after dividing the population into Asian and non-Asian groups, stromal and immune scores were independent of OS in Asian patients, but they were associated with OS in non-Asian patients. Kaplan–Meier survival analysis also showed that four DEGs (IGHM, IGLV1-51, IGLV2-8, and IGLV3-10) were significantly associated with OS in non-Asian patients but not in Asian patients.

IGHM was reported to be a stromal/immune-related differentially expressed gene in liver cancer [38]. Some immunoglobulin genes, such as IGHV, IGHV5-78, IGL, and IGKC, were expressed at low levels in HBV-induced HCC, but they were highly expressed in HCV-induced HCC [39]. One hundred and twenty distinct immune genes showed differential expression in HBV/HCV-induced HCC [39]. Polymorphisms in Th1 and Th2 cytokine genes are associated with the risk of HCC in Chinese individuals [40]. However, Th1 and Th2 genotypes did not majorly impact the incidence of HCC in the USA [41]. This evidence suggests that the difference in immune gene activation was due to different etiologies in HCC.

Variations in genetic mutations have also been found in different populations of HCC. For example, SALL-4, a novel oncofetal protein, is expressed in 47.7% of Chinese patients, but it is only expressed in 1.3% of Western patients [42,43]. The differences in genetic mutations may be due to etiology, environmental, or ethnic effects. The high heterogeneity of HCC could explain why the ESTIMATE algorithm analysis showed differences between the Asian and non-Asian groups.

Our CIBERSORT analysis showed that the composition of infiltrating immune cells was significantly different between Asian and non-Asian patients. Infiltrating immune cells and immune microenvironment subtypes were associated with clinical outcomes and immunotherapy responses [44]. It is important to classify the diversity of the immune context of the tumor microenvironment in HCC.

However, we have no evidence regarding whether the heterogeneity of the patient population (Asian and non-Asian groups) could be attributed to different viral etiology, environmental, or ethnic factors of HCC.

## 5. Conclusions

Because of the heterogeneity of HCC in terms of biology, etiology, and epidemiology, differences in clinical research and treatment approaches are needed. In this study, our data demonstrated that the ESTIMATE algorithm is suitable for the non-Asian HCC population but not for the Asian population. CIBERSORT analysis may be more suitable for Asian HCC populations. Racial disparities in multiethnic populations are notable. By parsing the subclasses of HCC, the ability to predict prognosis and guide therapeutic targets for potentially actionable HCC may be improved. 

Furthermore, with HBV vaccination and antiviral agents against HCV implementation, nonalcoholic fatty liver disease (NAFLD) has become a leading cause of liver carcinogenesis [29]. By 2030, the total NAFLD population is projected to increase by 18.3% in the United States and 29.1% in China [45]. NAFLD-associated HCC will remain a major challenge in the future. There is no FDA-approved drug for the effective treatment of NAFLD-HCC [46]. Therefore, classifying HCC is important for clinical research and treatment approaches. A better understanding of the etiology of HCC subtypes or in multiethnic populations will increase treatment options for HCC.

## Figures and Tables

**Table 1:** Clinical information of patients with HCC.

	Asian		Non-Asian		*p*-Value
	(*n* = 205)		(*n* = 154)		
Age (years)	55.42 ± 11.27		62.92 ± 13.65		<0.001
Body weight (kg)	62.59 ± 10.56		81.52 ± 20.92		<0.001
Gender					<0.001
Male	121	78.60%	120	58.50%	
Female	33	21.40%	85	41.50%	
Stage					0.498
Stage I	79	51.30%	88	42.90%	
Stage II	34	22.10%	50	24.40%	
Stage III	38	24.70%	44	21.50%	
Stage IV	1	0.60%	4	2.00%	
NA	2	1.30%	19	9.30%	
Survival status					0.008
Alive	119	58.00%	111	72.10%	
Dead	86	42.00%	43	27.90%	
Survival Time (months)	26.00 ± 22.76		27.43 ± 24.84		

**Table 2:** The Kaplan–Meier survival analysis results of five hub genes.

	*p*-Value		
Gene	All (*n* = 359)	Asian (*n* = 205)	Non-Asian (*n* = 154)
IGHM	0.012	0.263	0.015
IGKV4-1	0.026	0.186	0.068
IGLV1-51	0.013	0.440	0.020
IGLV2-8	0.008	0.655	0.002
IGLV3-10	0.004	0.127	0.018

**Table 3:** The Kaplan–Meier survival analysis results of infiltrating immune cell subsets.

	*p*-Value	
	Asian	Non-Asian
Naïve B cells	NA	NA
Memory B cells	0.041	0.574
Plasma cells	0.004	0.186
CD8 T cells	0.082	0.131
CD4 naïve T cells	0.816	0.136
Resting memory CD4 T cells	0.012	0.465
Activated memory CD4 T cells	<0.001	0.115
Follicular helper T cells	0.940	0.066
Tregs	0.882	0.566
Γ delta T cells	NA	NA
Resting NK cells	0.296	0.370
Activated NK cells	0.017	0.107
Monocytes	0.515	0.135
M0 Macrophages	0.072	0.082
M1 Macrophages	0.004	0.564
M2 Macrophages	0.084	0.387
Resting dendritic cells	0.049	0.766
Activated dendritic cells	0.892	0.041
Resting mast cells	0.041	0.766
Activated mast cells	0.380	0.578
Eosinophils	NA	NA
Neutrophils	<0.001	0.006

## Data Availability

The original data obtained from TCGA database could be provided via the email of corresponding author for academic research purpose only.

## Supplementary Materials

The following are available online at https://www.mdpi.com/article/10.3390/genes12081274/s1, Table S1: DEGs in immune score groups in non-Asian HCC; Table S2: DEGs in stromal score groups in non-Asian HCC; Table S3: DEGs in immune score groups in Asian HCC; Table S4: DEGs in stromal score groups in Asian HCC.
